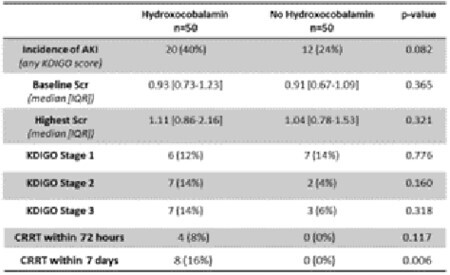# 511 Hydroxocobalamin Not the Clear Culprit of Nephrotoxicity After Cyanide Poisoning

**DOI:** 10.1093/jbcr/irae036.146

**Published:** 2024-04-17

**Authors:** Elise M Mann, Allison N Boyd, Todd A Walroth, Jessica Whitten, Allyson M McIntire, Serena Dine, Molly M Howell, Taylor Rhew, Brett C Hartman

**Affiliations:** UofL Health, Louisville, KY; Eskenazi Health, Zionsville, IN; Eskenazi Health, Indianapolis, IN; Indiana University, Indianapolis, IN; UofL Health, Louisville, KY; Eskenazi Health, Zionsville, IN; Eskenazi Health, Indianapolis, IN; Indiana University, Indianapolis, IN; UofL Health, Louisville, KY; Eskenazi Health, Zionsville, IN; Eskenazi Health, Indianapolis, IN; Indiana University, Indianapolis, IN; UofL Health, Louisville, KY; Eskenazi Health, Zionsville, IN; Eskenazi Health, Indianapolis, IN; Indiana University, Indianapolis, IN; UofL Health, Louisville, KY; Eskenazi Health, Zionsville, IN; Eskenazi Health, Indianapolis, IN; Indiana University, Indianapolis, IN; UofL Health, Louisville, KY; Eskenazi Health, Zionsville, IN; Eskenazi Health, Indianapolis, IN; Indiana University, Indianapolis, IN; UofL Health, Louisville, KY; Eskenazi Health, Zionsville, IN; Eskenazi Health, Indianapolis, IN; Indiana University, Indianapolis, IN; UofL Health, Louisville, KY; Eskenazi Health, Zionsville, IN; Eskenazi Health, Indianapolis, IN; Indiana University, Indianapolis, IN; UofL Health, Louisville, KY; Eskenazi Health, Zionsville, IN; Eskenazi Health, Indianapolis, IN; Indiana University, Indianapolis, IN

## Abstract

**Introduction:**

Inhalation injury (IHI) and cyanide poisoning are often observed in burn patients, but they may also present without cutaneous injury. If cyanide toxicity is suspected, the antidote, hydroxocobalamin, is indicated. Although a lifesaving drug, hydroxocobalamin has been associated with acute kidney injury (AKI) and oxalate nephropathy in recent literature. It remains unclear whether the renal issues observed after hydroxocobalamin administration are due to the drug itself or are influenced by other confounding variables. The objective of this study was to compare patients with IHI who either do or do not receive hydroxocobalamin and to assess whether there is a difference in incidence of nephrotoxicity.

**Methods:**

This single-center, retrospective, matched, cohort study included adults 18 years or older admitted to the Medical ICU or Burn ICU for at least 72 hours between 10/2016 and 12/2022 with a documented diagnosis of IHI. The primary outcome was the incidence of AKI, defined per KDIGO criteria, within 72 hours of admission. Secondary outcomes included initiation of continuous renal replacement therapy (CRRT) within 72 hours, CRRT within 7 days, length of stay, and vasopressor requirements. A power calculation was not performed a priori as this was a convenience sample of all eligible patients. A post hoc analysis was conducted to assess the correlation of AKI after hydroxocobalamin administration in those with IHI and cutaneous injury vs. IHI only.

**Results:**

A total of 100 patients, 50 who received hydroxocobalamin and 50 who did not, were matched based on TBSA and age and included in the analysis. Primary and secondary outcomes are included in Table 1 and Table 2. The was no difference in the incidence of AKI between the two groups. There were no differences in length of stay, Parkland Formula use, vasopressor requirement, or inpatient administration of nephrotoxic agents. Those who received hydroxocobalamin were found to have a higher median grade of IHI and had a higher incidence of CRRT initiation at 7 days. In the post-hoc analysis, there was no difference in AKI after hydroxocobalamin administration in those with IHI and cutaneous injury vs. IHI only.

**Conclusions:**

This study did not demonstrate a difference in the incidence of AKI in patients with IHI who did or did not receive hydroxocobalamin. This outcome was not changed by the presence or lack of concurrent cutaneous injury.

**Applicability of Research to Practice:**

To our knowledge, this is the first study that was controlled for hydroxocobalamin administration and evaluated clinical outcomes in both burn and non-burn IHI patients. Our findings differ from previous literature and suggest that hydroxocobalamin alone is not the clear culprit of nephrotoxicity in patients with IHI.